# A natural sustained-intestinal release formulation of red chili pepper extracted capsaicinoids (Capsifen®) safely modulates energy balance and endurance performance: a randomized, double-blind, placebo-controlled study

**DOI:** 10.3389/fnut.2024.1348328

**Published:** 2024-03-20

**Authors:** N. Roopashree, Das S. Syam, I. M. Krishnakumar, K. N. Mala, Bradley S. Fleenor, Jestin Thomas

**Affiliations:** ^1^BGS Global Institute of Medical Sciences, Bangalore, Karnataka, India; ^2^Akay Natural Ingredients Ltd, Kochi, Kerala, India; ^3^Sri Rama Hospital, Bangalore, Karnataka, India; ^4^DeBusk College of Osteopathic Medicine, Lincoln Memorial University, Harrogate, TN, United States; ^5^Leads Clinical Research and Bio Services Private Limited, Bangalore, Karnataka, India

**Keywords:** capsaicin, thermogenesis, Capsifen, endurance, energy expenditure, FenuMat, respiratory quotient

## Abstract

**Introduction:**

Overweight and obesity are major public health concerns, with a sharp increase in prevalence over the last few decades. The primary cause is an imbalance between calorie intake and expenditure due to a rise in calorie-rich processed food and reduced physical activity. Energy balance in humans involves complex processes including thermogenesis, a crucial factor in regulating energy expenditure.

**Methods:**

In this randomized, double-blinded, placebo-controlled three-arm three-sequence study, we investigated the efficacy of Capsifen® (CapF), a pungency-masked sustained-intestinal release formulation of red chili extract, on energy expenditure, fat oxidation, and endurance using the Quark C-PET system in healthy overweight participants, with and without exercise. In the study, 105 healthy participants were randomized to receive either placebo, CapF 100 mg/day, or CapF 200 mg/day for 28 days.

**Results:**

CapF demonstrated a dose-dependent response to increased energy expenditure and fatty acid oxidation with a concomitant reduction in body weight. Both CapF 100 and CapF 200 also increased the time to exhaustion.

**Discussion:**

These results demonstrate the plausible efficacy of CapF in energy expenditure and physical performance in otherwise healthy adults who have a high body mass index.

**Clinical trial registration:**

https://ctri.nic.in/Clinicaltrials/pmaindet2.php?EncHid=MjQzNTg=&Enc=&userName=CTRI/2018/04/013157 dated 04 October 2018.

## 1 Introduction

Obesity is a rapidly growing public health problem of significant concern. Over the past three decades, countries worldwide have experienced a 2- to 3-fold increase in the prevalence of obesity (1). In 2019, an estimated 5 million deaths worldwide were linked to obesity (2), and approximately one in three adults and one in six children in the United States were reported as obese (1). The primary cause of overweight and obesity is the imbalance between energy/calorie intake and expenditure. Increased consumption of calorie-dense, processed foods, and lack of sufficient physical activity have contributed to this imbalance (3). Modest adjustments to diet and physical activity, specifically reducing calorie intake and enhancing calorie expenditure, can effectively reduce obesity. Such minor changes are more feasible and sustainable in the long term than radical dietary or behavioral modifications (4, 5).

Maintaining a balance between energy intake and energy expenditure is a complex process. This balance is crucial for keeping the energy stores stable over time, following the principle of energy conservation (6). Energy expenditure consists of three components: resting energy expenditure (energy needed for basic bodily functions at rest), activity-induced energy expenditure (energy associated with physical activity), and diet-induced energy expenditure (energy used in digestion and metabolism). Diet-induced energy expenditure is approximately 10% of the total energy expenditure in normally active individuals (7), and it involves thermogenesis, a key factor in regulating temperature and energy balance (8, 9). Thermogenesis can be adaptive, responding to environmental changes, or facultative, triggered by eating (10, 11). Under obesity, around half of the weight loss was identified to be due to adaptive thermogenesis, which can be activated by specific food ingredients (12, 13). The major sites of thermogenesis are brown adipose tissues (BAT) and skeletal muscles (14). The average amount of BAT usually ranges from 0.02 to 300 g, which constitutes less than 0.5% of the total human body mass (15). Currently, there is a growing interest in exploring ways to modulate diet-induced thermogenesis to facilitate weight loss or maintaining a healthy body weight.

Several dietary spices and herbs, including red chili pepper, ginger, black pepper, long pepper, cinnamon, garcinia cambogia, yerba mate, green coffee, green tea, bitter orange, and guarana, have been shown to possess thermogenic effects or promote EE (8). Among different botanicals, red chili pepper stands out due to its remarkable spiciness (pungency). Furthermore, its medicinal properties are highly regarded, encompassing anti-inflammatory, anti-analgesic, anti-diabetic, anti-lipidemic, and anti-cancer effects in addition to the anti-obesity effects (16, 17). Several studies have demonstrated thermogenic, lipolytic, appetite-suppressing, and weight loss properties of red chilies (18, 19). However, the sensory burn and gastrointestinal side effects associated with red chili use remain a major challenge for its clinical use.

We have recently developed a taste-masked, sustained-intestinal release beadlet formulation of red chili pepper extract containing 2% capsaicinoids [a sum of capsaicin, dihydrocapsaicin (DC), and nordihydrocapsaicin (NDC)]. This was achieved using a proprietary hydrogel technology known as FenuMat® (20). The microbeadlets known as Capsifen® (CapF) are bioavailable and safe at dosages of 200 mg/day in individuals with obesity and helped to reduce body weight following 28 days of supplementation (21). However, it is unknown whether lower dosages are efficacious for supporting weight management. Therefore, the primary objective of this study was to examine the effects of a low dose (100 mg/day) and a high dose (200 mg/day) of CapF over a period of 28 days on energy expenditure, fat oxidation, and endurance. These parameters were evaluated using the Quark C-PET system, which served as the validated assessment tool during cardiopulmonary exercise tests (CPET) (22). The evaluation was conducted on healthy subjects, both with and without exercise.

## 2 Materials and methods

### 2.1 Study design

**Figure 1:**
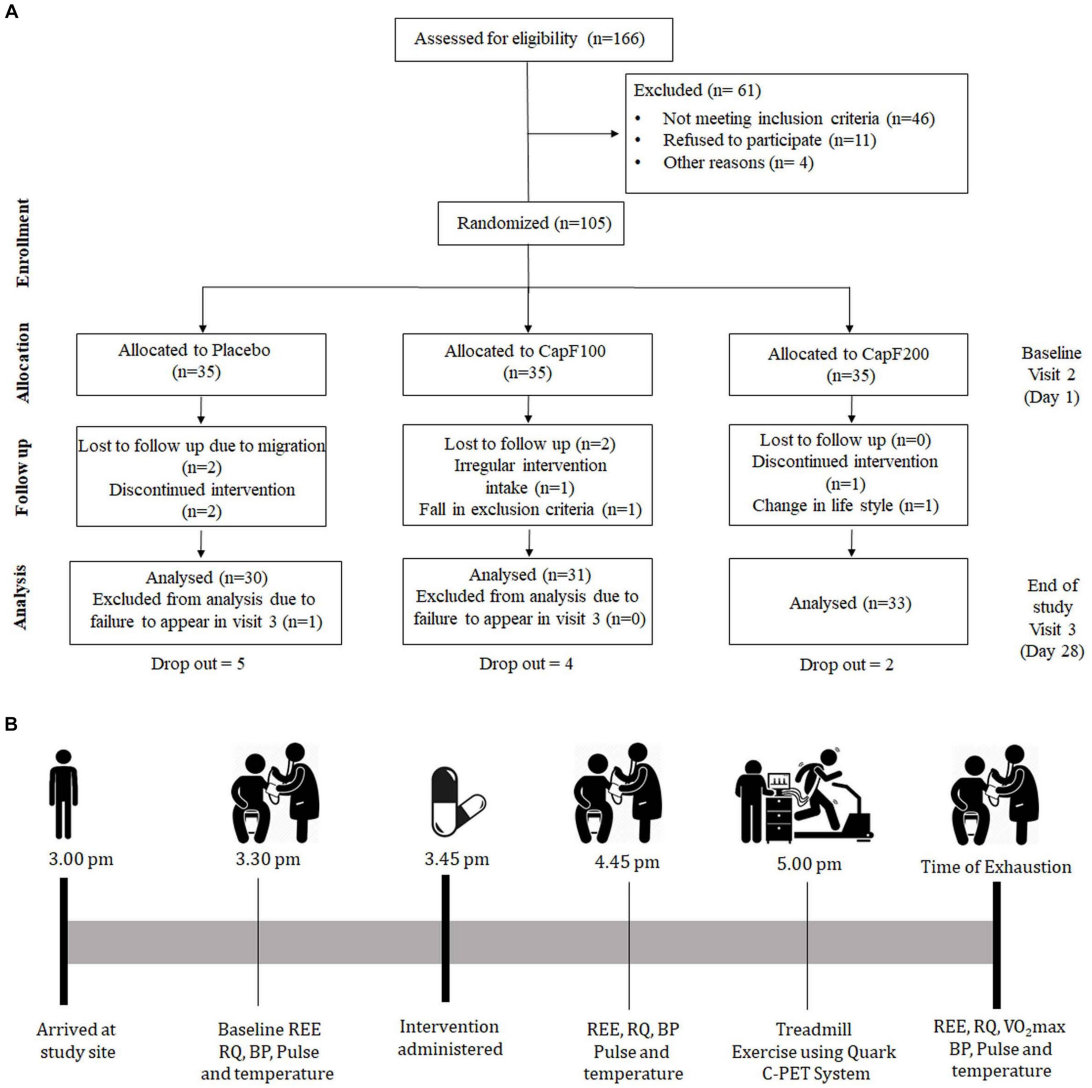
**(A)** CONSORT diagram illustrating the study design; **(B)** evaluation scheme on visit 2 and visit 3.

The study involved a randomized placebo-controlled, three-arm, three-sequence, comparative as summarized in Figure 1 and conducted at Sri Rama Hospital, Bangalore, India, under the supervision of a registered medical practitioner in accordance with the Declaration of Helsinki. The institutional ethical committee reviewed and approved the protocol and the study was registered with the Clinical Trial Registry of India (CTRI/2018/04/013157). A written informed consent ensuring their awareness of the study details and voluntary participation was also obtained from all the participants prior to the commencement of the study.

### 2.2 Subjects

Healthy adults (males and females) aged 21–45 years with a body mass index (BMI) ≥ 25 kg/m^2^ were recruited for this study. A total of 166 healthy volunteers were screened, and 105 participants meeting the inclusion and exclusion criteria were enrolled in the study. The major inclusion/exclusion criteria are provided in Table 1. Those who expressed interest were provided with comprehensive information about the trial and an online screening questionnaire. This questionnaire assessed general health, medication usage, alcohol, and drug consumption (including nicotine), supplement and vitamin intake, and pregnancy or breastfeeding status. Individuals who were considered eligible based on the screening were invited to the study site for further screening.

**Table 1:** Major inclusion and exclusion criteria of the study.

Inclusion criteria	Exclusion criteria
Male and female subjects aged from 21 to 45 years of age	Subjects suffering from any chronic health conditions (e.g., diabetes, thyroid issues, hypertension, chronic renal failure, heart, and liver disease)
Healthy subjects with elevated lipid profile	The subject is visually challenged or currently suffers from a sleep disorder and/or has a known history of (or is currently being treated for) clinical depression, eating disorder(s), or any other psychiatric condition(s), which in the opinion of the investigator, might put the subject at risk and/or confound the results of the study.
Has BMI ≥ 25 kg/m^2^	History of cardiac health issues, chronic metabolic diseases, drug abuse, smoking, abuse/addiction to alcohol, endocrine abnormalities. Including stable thyroid disease, any major surgery, including cardiovascular surgery and bariatric surgery
Non-smoker	History of allergic reaction to herbal products or any component of the study product
Not been on any concomitant medication for the last 6 months.	Subjects on any other medical or clinical investigation or any nutrition supplements and dietary regime
Females of childbearing age, agree to use approved birth control methods during the study, and have a negative urine pregnancy test (UPT) at screening	Known HIV/hepatitis B positive or any other immunocompromised state
Subject understands the study procedures, is ready to comply, and provides a signed informed consent form (ICF) to participate in the study	Currently participating or having participated in another clinical trial during the last 3 months prior to the beginning of this study
	Any additional condition(s) that in the Investigators opinion would warrant exclusion from the study or prevent the subject from completing the study

### 2.3 Intervention

Capsicum extracts infused in fenugreek galactomannan (mucilage) beadlets, known as Capsifen® (CapF), were standardized to contain 2.1% total capsaicinoids [comprising capsaicin, dihydrocapsaicin (DC), and nordihydrocapsaicin (NDC)]. Capsaicinoid content was estimated by a validated HPLC method (23), utilizing a Shimadzu LC 20 AT instrument equipped with an M20A photodiode array detector (PDA; Shimadzu Analytical Private Limited, Mumbai, India). In this study, identical hard shell gelatin capsules containing placebo (microcrystalline cellulose) 100 mg × 1 capsule/day, CapF 100 mg × 1 capsule/day, and CapF 200 mg × 1 capsule/ day were provided in airtight amber-colored bottles of similar size and shape. Each bottle contained 35 capsules, and the subjects were advised to consume the respective supplement after their major meal.

### 2.4 Randomization and blinding

Subjects were randomized during visit 1, using computer-generated randomization codes, into one of three arms to receive a placebo, CapF 100, or CapF 200 for 28 days. All capsules were identical in size, shape, and appearance. The master randomization list prepared by an independent statistician was handed over to the pharmacist for the purpose of double blinding. The investigator and other faculties involved in the study were not aware of allocation or intervention details.

### 2.5 Study protocol

The primary objective of the study involved measuring EE and respiratory quotient (RQ) upon the supplementation of CapF. The REE is the collective energy required by the body cells to maintain post-absorptive homeostatic functions at rest (24). The RQ, also known as the respiratory ratio, is defined as the volume of carbon dioxide released over the volume of oxygen inhaled during respiration (25).

We conducted a cardiopulmonary exercise test (C-PET) using a metabolic cart (Quark C-PET Systems, COSMED, Italy) and measured EE and RQ for 10 min. Quark C-PET system is a validated system used for evaluating energy metabolism (22, 26). To measure these parameters, subjects wore a headgear attached to a mask which allowed metabolic assessment by measuring the volume of O_2_ inhaled and CO_2_ exhaled during exercise. This protocol required the participant to begin exercise at a self-selected speed between 3 and 6 km/h. The self-selected speed was maintained, while the treadmill elevation increased by 2% every 2 min during the test. Time to exhaustion was determined as the time that the subject could no longer maintain exercise intensity and/or reached volitional exhaustion. Data were acquired continuously during the exercise protocol, and the maximal RQ was obtained upon volitional exhaustion. Resting energy expenditure (REE—i.e., energy expenditure under resting conditions) was also obtained from the Quark C-PET System.

In a typical protocol, there were a total of three visits: visit 1, screening and randomization; visit 2, day 1, baseline; visit 3, day 28 or the end of study (Figures 1A,B). At visit 2, the baseline characteristics of the participants [demographic characteristics, health conditions, physical examination, anthropometric measurements, blood pressure, measurements of energy expenditure (EE) and respiratory quotient (RQ)] were measured. On visit 3, all the above-mentioned parameters were analyzed the same as on visit 2.

All participants were instructed to arrive at the study site on day 1 (visit 2) at approximately 3 pm and were allowed to rest for 15 to 20 min at their convenience. They were provided with a bottle containing 35 capsules and were instructed to take the assigned intervention (placebo, CapF 100, or CapF 200) daily for 28 days with 200 ± 20 mL water. Prior to supplementation and 1 h post-supplementation, EE, RQ, blood pressure, and heart rate were measured. On the completion of resting measures, a maximal cardiopulmonary exercise test (CPET) was performed to determine oxygen utilization. All participants were advised to maintain their regular physical activities. Adherence to the study protocol was ensured with weekly telephonic conversation, and the binding to the protocol was estimated by count-pill strategy. On day 28 (visit 3), the subjects returned to the study site and repeated the aforementioned measurements.

Participants were placed on a standardized Indian food diet (containing carbohydrates, proteins, and fat) comprising of rice, vegetables, and eggs for breakfast (protein: 27–30%; fat 20–24%; carbohydrate 35%–40%; energy 400–500 calories); rice, chicken, and vegetables for lunch (protein 27–30%; fat 20%–24%; carbohydrate 35%–40%; energy 400–500 calories); wheat, chicken, and vegetables for dinner (protein 27%–30%; fat 20%–24%; carbohydrate 25%–35%; energy 400–500 calories) during the duration of the study. There was no restriction on drinking water.

### 2.6 Clinical assessment

All measurements were performed at baseline (day 1) and at the end of the study (day 28). Anthropometric indexes, including weight and height, were assessed with light clothing and barefoot to the nearest 0.1 kg and 0.5 cm, respectively, using a scale with a stadiometer. Demographic characteristics, health history, and physical examination were performed in the presence of a registered medical practitioner. Blood pressure was measured using a digital device (IntelliSense®, Omron, Japan).

### 2.7 Statistical analysis

Based on a previous study, the sample size was calculated using G-Power (20). The sample size was estimated as 35 in each arm while having 80% power with an alpha of 0.05 and a dropout rate of 20%. All the statistical analyses were performed using SPSS software version 28.0 (SPSS Inc., Chicago, IL, United States). Data are represented as mean ± standard deviation (SD). A 2 × 3 repeated-measures ANOVA with post-hoc analysis was used to evaluate the statistical significance between placebo and CapF groups. A *p*-value < 0.05 was considered statistically significant.

## 3 Results

Baseline demographic data of the participants demonstrate no intra- or inter-group differences in heart rate and systolic and diastolic blood pressure (*p* > 0.05; Table 2). Body weight was significantly reduced in the CapF 100 and CapF 200 groups, but not in the placebo group. The reduction in BMI in the CapF 100 and CapF 200 groups was also statistically significant (*p* < 0.05).

**Table 2:** Changes in subjective parameters upon supplementation of CapF at 100 and 200 mg/day with respect to placebo and baseline.

Parameters	Placebo (*n* = 30)		CapF 100 (*n* = 31)		CapF 200 (*n* = 33)		*p*-values	
	Baseline	Week 4	Baseline	Week 4	Baseline	Week 4	Time	Group
Age, years	28.00 ± 4.30		32.80 ± 4.00		33.10 ± 4.20		---	
Males/females	27/8		26/9		24/11		---	
Weight, kg	74.3 ± 5.1	76.5 ± 5.3	75.1 ± 5.4	71.5 ± 5.2	75.3 ± 5.3	70.7 ± 5.1	0.001	0.027
BMI, kg/m^2^	28 ± 1.3	28.9 ± 1.3	27.6 ± 0.7	26.4 ± 1	28.3 ± 0.9	26.6 ± 0.9	0.001	0.001
Heart rate, beats/min	71 ± 4	73 ± 4	72 ± 2	71 ± 3	72 ± 3	71 ± 3	0.72	0.93
Systolic blood pressure, mm Hg	119 ± 2	120 ± 2	119 ± 3	118 ± 2	119 ± 2	120 ± 3	0.21	0.16
Diastolic blood pressure, mm Hg	77 ± 5	76 ± 5	76 ± 5	78 ± 6	74 ± 6	77 ± 5	0.52	0.15

### 3.1 Effect on energy expenditure

#### 3.1.1 Under resting conditions after exercise

A 2 × 3 repeated-measures ANOVA was performed to compare the effect of CapF 100 and CapF 200 on EE-R. There was a statistically significant difference in EE-R between the placebo and treated groups [*F*(1,2) = 122.90, *p* = 0.026].

**Figure 2:**
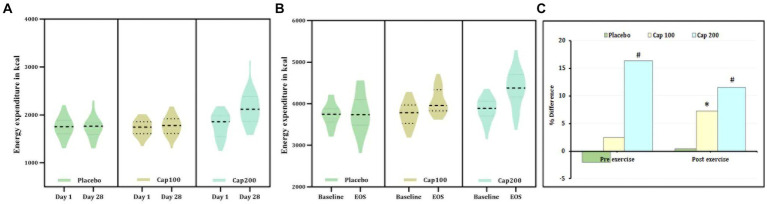
Violin plot showing the acute and chronic effects of CapF on energy expenditure at rest and at 1 h following the maximal exercise for three groups (placebo, CapF 100, and CapF 200). The violin plot outlines demonstrate kernel probability density. The width of the shaded area shows the distribution of the data. The thick dotted line shows the median, and the thin dotted lines represent quartiles. **(A)** Energy expenditure without exercise. The enhancement in energy expenditure under resting conditions (ΔEE-R) was >11-fold in CapF 100 mg/day compared to placebo, whereas CapF 200 showed 300 kcal ΔEE-R, which was 16-fold higher than placebo. **(B)** Energy expenditure 1 h after maximal exercise. The enhancement in exercise-induced energy expenditure (ΔEEE) was 11.08 kcal in the placebo group and 277.14 kcal in the 100 mg/day group, respectively, which was ~25-fold higher than the placebo. CapF 200 supplementation resulted in 330.8 kcal in ΔEEE, which was 30-fold higher than placebo. The values are expressed as mean ± SD. **(C)** Percentage difference based on baseline values for Placebo, CapF 100, and CapF 200. The symbols “*” and “#” in the bar diagram indicate significant differences at *p* < 0.05 compared to placebo.

Analysis of within-subject effect showed a significant increase in EE-R for both CapF 100 (*p* < 0.001) and CapF 200 (*p* < 0.001) compared to baseline, but the placebo did not show any change (Figure 2A). This corresponded to a 195-kcal increase for CapF 100, 300 kcal for CapF 200, and − 18.2 kcal for placebo. The observed changes in EE-R (∆EE-R) for the CapF 100 and CapF 200 groups were 11-fold and 16-fold increase compared to placebo.

#### 3.1.2 Effect on exercise-induced energy expenditure

The results of ANOVA revealed that there was a statistically significant interaction between the effects of placebo, CapF groups, and EEE [*F*(1, 2) = 7.12, *p* < 0.001].

The observed energy expenditure after exercise (on resting) is mentioned as exercise-induced energy expenditure. In both CapF 100 (*p* = 0.001) and CapF 200 groups (*p* < 0.001), the within-group effect of EEE demonstrated a significant rise compared to baseline, while the placebo group exhibited no significant change (Figure 2B). The relative change in the outcome (ΔEEE) representing the difference between the baseline and the end of the study was 277.14 kcal for CapF 100 (*p* = 0.001) and 330.8 kcal (*p* < 0.001) for the CapF 200 group, which were 25-fold and 30-fold higher, respectively, than the ΔEEE for the placebo (Figure 2C).

### 3.2 Effect on respiratory quotient

#### 3.2.1 Effect on resting RQ

**Figure 3:**
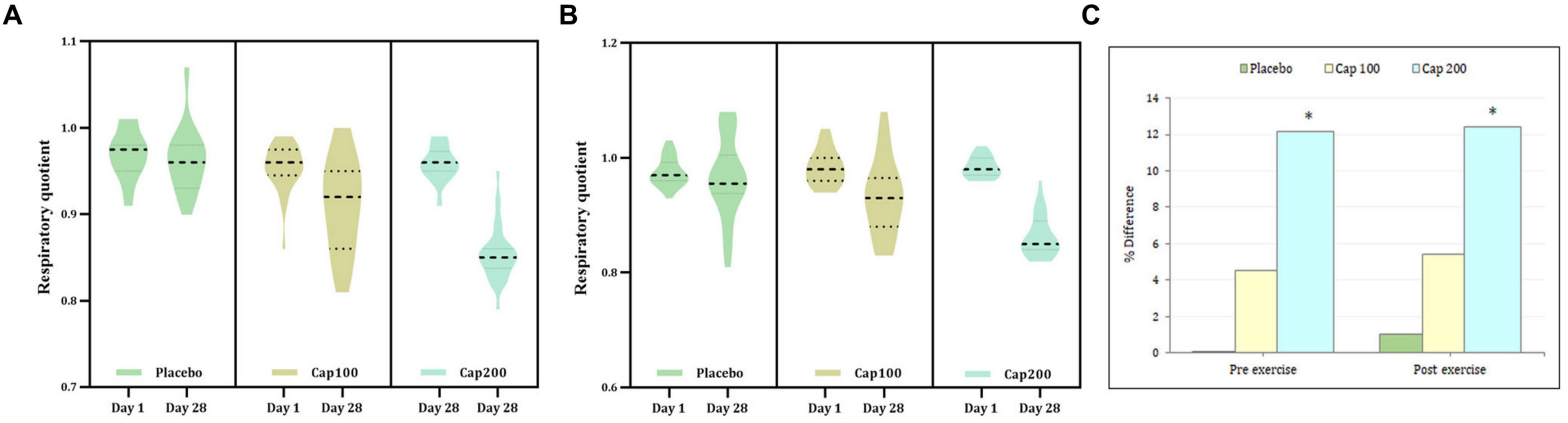
Violin plot showing the acute and chronic effects of CapF on respiratory quotient (RQ) at rest and at 1 h following the maximal exercise for three groups (placebo, CapF 100 and CapF 200). The violin plot outlines demonstrate kernel probability density. The width of the shaded area shows the distribution of the data. The thick dotted line shows the median, and thin dotted lines represent quartiles. **(A)** Treatment vs. time of RQ before exercise showed significant reduction at doses of 100 mg/day (*p* < 0.001) and 200 mg/day (*p* < 0.001) respectively at the end of study. The percentage of reduction were 4.2 and 11.45% respectively; **(B)** Treatment vs. time of RQ after exercise showed significant reduction at doses of 100 mg/day (*p* < 0.001) and 200 mg/day (*p* = 0.001) respectively at the end of study. The percentage of reduction were 5.10 and 12.24% respectively. The values are expressed as mean ± SD. **(C)** Percentage difference based on baseline values for placebo, CapF 100 and CapF 200. The symbol “*” in the bar diagram indicate significant difference at *p* < 0.05 compared to placebo.

The respiratory quotient was measured before and after exercise. In this study, the simple main-effect analysis of resting respiratory quotient (RRQ) for the CapF 100 showed 4.2% (*p* < 0.001) and the CapF 200 showed a 11.45% reduction (*p* < 0.001) at the end of the study when compared to baseline, while the placebo showed no significant change [*p* > 0.05; Figure 3A; *F*(2,2) = 11.06; *p* < 0.001].

Pairwise comparison of between-group effects at the end of the study showed a significant reduction in RRQ for CapF 100 (4.16%; *p* = 0.038) and CapF 200 (11.4%; *p* < 0.001) respectively compared to placebo. The CapF 200 showed a significant reduction (*p* < 0.001) when compared with the CapF 100.

#### 3.2.2 Effect on exercise-induced respiratory quotient

Within-subject main-effect comparison of exercise-induced respiratory quotient (ERQ) showed a significant reduction for the CapF 100 (5.1%; *p* = 0.001) and a 12.24% reduction in the CapF 200 (*p* < 0.001), respectively, compared to baseline (Figure 3B). However, there was no significant change (*p* > 0.05) for the placebo [*F*(1,1) = 52.70; *p* = 0.001].

Between-group effect (the CapF 100 group vs. the placebo group) showed no significant reduction (3.12%; *p* > 0.05) for the CapF 100. However, the CapF 200 showed a significant reduction (10.41%; *p* < 0.001) compared with the placebo. The CapF 200 showed more reduction than the CapF 100 in ERQ, and the percentage of reduction was statistically significant (*p* < 0.001; Figure 3C).

### 3.3 Effect on fat oxidation

#### 3.3.1 Resting fat oxidation

**Figure 4:**
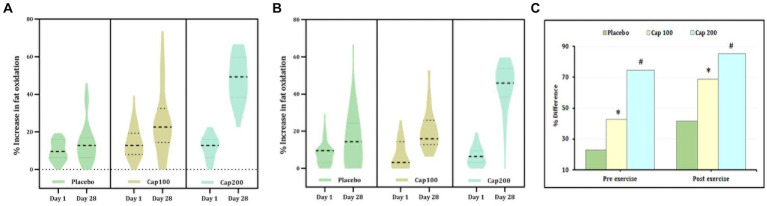
Violin plot showing the acute and chronic effects of CapF on fat oxidation (FO) at rest and at 1 h following the maximal exercise for three groups (placebo, CapF 100, and CapF 200). The violin plot outlines demonstrate kernel probability density. The width of the shaded area shows the distribution of the data. The thick dotted line shows the median, and the thin dotted lines represent quartiles. **(A)** Fat oxidation without exercise. The enhancement in fat oxidation was 77.25% in CapF 100 compared to placebo. However, the 200 mg/day supplemented group showed 292.25%, which was 10-fold higher than in placebo. Statistical analysis showed a significant increase in % fat oxidation (*p* < 0.001) in both 100 mg and 200 mg dosages. **(B)** Fat oxidation following maximal exercise. Statistical analysis showed a significant increase in % fat oxidation (*p* < 0.001) in both 100 mg and 200 mg dosages. **(C)** Percentage difference based on baseline values for placebo, CapF 100, and CapF 200. The symbols “*” and “#” in the bar diagram indicate significant differences at *p* < 0.05 compared to placebo.

Fat oxidation was calculated from RQ under conditions of rest, 1 h post-administration following exercise. The percentage increase in fat oxidation was 74.84% with the CapF 100 and 292.25% with the CapF 200, indicating a 3-fold increase in fat oxidation when the dosage was doubled (Figure 4A). This increase was statistically significant [*F*(1,2) = 37.69; *p* < 0.001]. At the end of the study, between-group effects resulted in a 77.25% increase in fat oxidation for the CapF 100 (*p* = 0.038) and a 231.7% increase for the CapF 200 (*p* < 0.001) compared to the placebo. Additionally, the comparison between the CapF 100 vs. the CapF 200 revealed a significant increase (*p* < 0.001) for the CapF 200 in RFO.

#### 3.3.2 Exercise-induced fat oxidation

The main effect observed in fat oxidation after exercise did not show a significant difference between the placebo and the CapF 100 or CapF 200 on day 1. However, supplementation with CapF 100 and CapF 200 resulted in a significant increase in fat oxidation by the end of the study [Figure 4B; *F*(1,2) = 28.78; *p* < 0.001].

The relative percentage increase upon between-group effects was 220% compared to baseline and 36% compared to the placebo. The CapF 200 group showed a 591% increase compared to baseline values (Figures 4B,C) and 185% compared to the placebo. The CapF 100 group vs. the CapF 200 group showed a significant difference (*p* = 0.001) in EFO, and the increase is high in the CapF 200.

### 3.4 Effect on endurance

**Figure 5:**
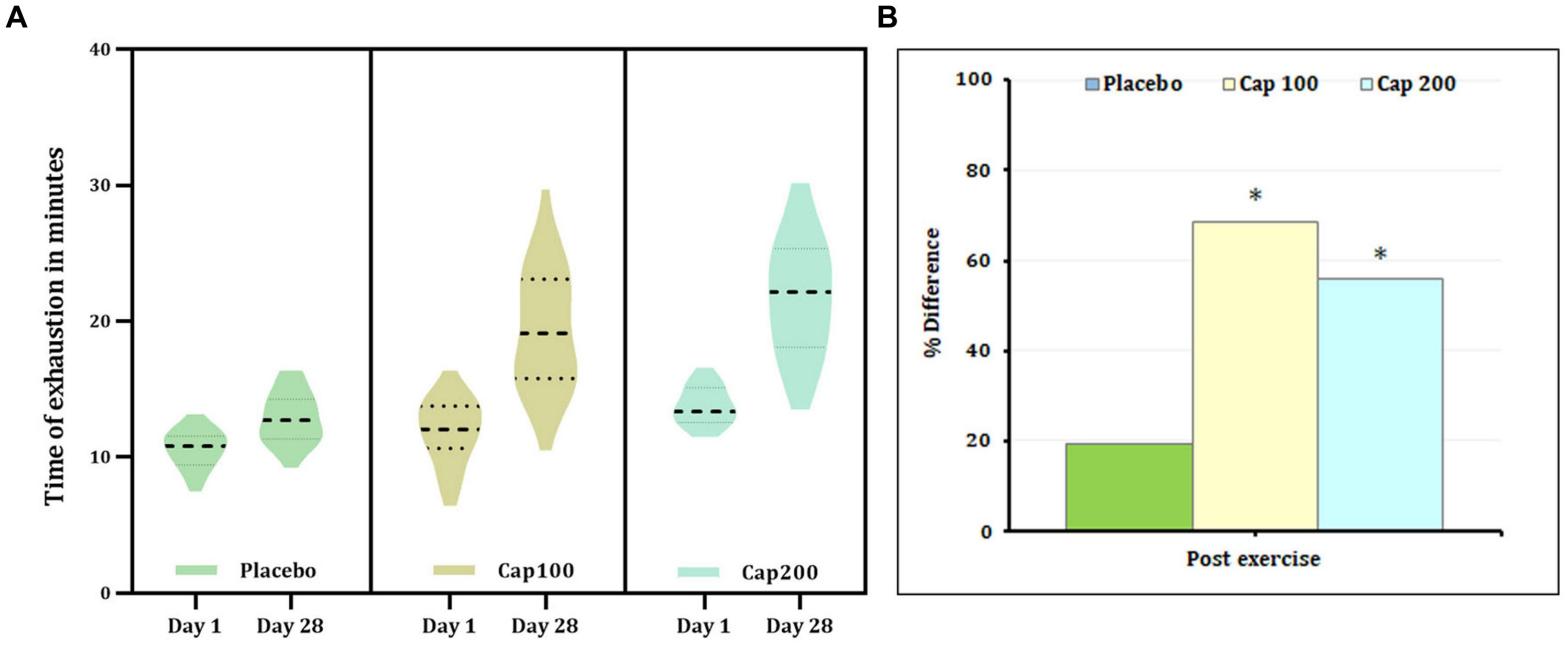
**(A)** Violin plot showing the acute and chronic effects of CapF on the time to exhaustion at rest and at 1 h following the maximal exercise for three groups (placebo, CapF 100, and CapF 200). The violin plot outlines demonstrate kernel probability density. The width of the shaded area shows the distribution of the data. The thick dotted line shows the median, and thin dotted lines represent quartiles. The results revealed significant increase in time of exhaustion compared to baseline upon supplementation with CapF 100 and CapF 200 (*p* < 0.001). The increase in CapF 100 is 68.52% (*p* < 0.001) and that of CapF 200 is 56.13% (*p* < 0.001). No significant change was observed in placebo (*p* > 0.05). **(B)** Percentage difference based on baseline values for placebo, CapF 100 and CapF 200. The symbol “*” in the bar diagram indicate significant difference at *p* < 0.05 compared to placebo.

The results of 2 × 3 repeated-measures ANOVA revealed a significant increase in time of exhaustion compared to baseline upon supplementation with CapF 100 and CapF 200 [*F*(1,2) = 21.41; *p* < 0.001]. The increase in CapF 100 is 68.52% (*p* < 0.001) and that of CapF 200 is 56.13% (*p* < 0.001; Figure 5). No significant change was observed in the placebo (*p* > 0.05).

Analysis of between-group effects at the end of the study revealed a 48.98% increase in the CapF 100 compared to the placebo, demonstrating statistical significance (*p* < 0.001). Additionally, the comparison between the placebo and the CapF 200 groups showed a significant 74.41% increase. Moreover, there was a significant increase in endurance for CapF 200 (*p* < 0.001) compared to CapF 100.

### 3.5 Adverse events

In total, four subjects in the CapF 200 group reported abdominal pain in the first 3 days, after intervention started; 2 subjects had diarrhea for the initial days and were able to control it in 1 week. Cold milk was administered to resolve the symptoms. No other adverse events were reported in either the CapF 100 or the CapF 200 group.

## 4 Discussion

The present study demonstrates notable dose-dependent enhancements of energy expenditure and improvements in exercise performance with CapF, a pungency-masked sustained-intestinal release beadlets of pungent chili extract with fenugreek mucilage. Specifically, energy expenditure (both resting and exercise-induced), fat oxidation, and endurance were enhanced, without significant adverse events such as heart palpitations, abdominal pain, or nausea. Moreover, the respiratory quotient, a measure of substrate oxidation, was also found to be decreased in the CapF groups compared to placebo. Thus, our results indicate the safety and efficacy of CapF-red chili pepper extract formulation. A previous pilot study on overweight subjects had also reported the safety and efficacy of CapF on body weight, BMI, and waist/hip ratio when supplemented at 200 mg/day for 28 days (21). Moreover, animal studies have established the sustained-intestinal delivery, systemic absorption, and safety (acute and subchronic toxicity) of CapF (27).

Herein, we report the first randomized, double-blind, placebo-controlled study to investigate the influence of CapF on energy expenditure and respiratory quotient. High-calorie intake and low energy expenditure are the most common characteristics of a sedentary lifestyle leading to obesity (28). Dietary components are key to support low-calorie intake and enhanced energy expenditure. Red chili pepper consumption has been shown to have a positive influence on food intake and energy expenditure (29). Preclinical studies have substantiated the thermogenic and lipolytic effects of red chili pepper (30). Despite these encouraging effects, clinical studies have very often provided mixed results. The main challenge in clinical settings was the consumption of a physiologically relevant dosage of pungent red chili pepper extract for the systemic absorption of its bioactive capsaicinoids. We hypothesized that CapF would provide significant functional benefits due to its sustained intestinal release and systemic absorption as reported previously (20, 27). Moreover, 2 and 4 mg capsaicinoids per day, the dosage used in this study, has also been reported to have thermogenic effect in humans (31).

Our study indicates a substantial increase in both resting and exercise-induced energy expenditure (EE-R and EEE) when CapF was consumed at 100 and 200 mg/day (2 and 4 mg capsaicinoids) for 28 days. While the CapF 100 group showed an 11-fold increase in EE-R and a 25-fold increase in EEE, the CapF 200 group showed a 16-fold and 30-fold increase, respectively, compared to their baseline values. The relative change in the mean difference of outcomes observed in the CapF 200 group was significantly higher than the CapF 100 demonstrating a dose-dependent response. Previous human interventions with capsaicinoids have also reported enhanced energy expenditure (31, 32), supporting the current findings.

Thermogenic effects, elevation in energy expenditure and core body and skin temperature, of pungent chilies have been reported (33–37). It is known that a 10% to 13% increase in metabolic rate can increase the core body temperature by 1° centigrade, which would lead to an increase in caloric expenditure of 100 to 130 Kcal/day (38). We observed that the consumption of CapF is increasing the body temperature by approximately 1 to 2°C when measured in a temperature- and humidity-controlled environment.

A significant reduction of the respiratory quotient (RQ) in the CapF groups, under both resting and exercise conditions, indicates enhanced fatty acid metabolism (31). Furthermore, our findings demonstrate a dose-dependent response in fatty acid metabolism with higher CapF doses. Fat oxidation can be assessed clinically from the RQ; higher RQ values are indicative of low-fat oxidation and high carbohydrate oxidation (39). The observed reduction in RQ indicates increased fatty acid oxidation compared to placebo. Fatty acid oxidation can decrease plasma free fatty acids (FFAs), which otherwise can have various negative effects, including elevation in insulin resistance, non-alcoholic fatty liver disease (NAFLD), development of type II diabetes, and related comorbidities such as cardiovascular disease (CVD) (40). Thus, enhancing fatty acid oxidation with CapF may provide for a novel dietary approach to aid with weight management and other comorbidity as mentioned above.

Yet another important parameter measured in the present study was the time to exhaustion, which showed a significant increase for CapF groups indicating enhanced endurance performance (26). Recently, de Freitas et al. showed performance improvements in a 1,500-m running time trial, high-intensity intermittent exercise, and resistance training when supplemented with a higher dose (12 mg) of capsaicinoids (41–43). The influence of capsaicin on endurance performance may be attributed to its ability to activate the transient receptor potential vanilloid 1 (TRPV1) channel on sensory neurons, thereby improving ATP production, vascular function, and fatigue resistance (44). Additional studies are needed to elucidate the mechanisms for the performance-enhancing effects of CapF.

Increase in heart rate, palpitations, sweating, and abdominal pain are generally considered as the major side effects of capsaicin, whereas nausea, vomiting, dizziness, dysgeusia, headaches, and hypoesthesia are minor adverse events found to be associated with pungent red chili peppers or its extracts (45). We observed no significant change in heart rate or blood pressure after 28 days of treatment with both CapF 100 and CapF 200 indicating its primary safety. A few volunteers did experience abdominal pain, burning sensations, and diarrhea in the initial 4 to 5 days while consuming the supplement with an empty stomach. It is advised to avoid red chili pepper extract consumption with an empty stomach. No volunteers reported heart palpitations indicating its slow release.

Nevertheless, the study has certain limitations. Lack of detailed information on day-to-day food intake and physical activities, the lack of baseline resting energy expenditure data measured under fasting conditions, and the use of a self-selected treadmill speed include the major limitations physical activities, the lack of baseline resting energy expenditure data measured under fasting conditions, and the use of a self-selected treadmill speed include the major limitations. Additionally, measurement of thermogenesis and respective molecular markers would have added further value to the study. Future research addressing these factors, with a larger population, is recommended.

## 5 Conclusion

Pungent red chili pepper extracts and their principal bioactive, capsaicinoids, have been found to offer significant benefits for body physiology, especially under conditions of overweight and obesity by increasing thermogenesis via stimulating various physiological responses. However, consuming capsaicinoids at physiologically relevant dosages with systemic absorption is a primary challenge, due to the extreme pungency of chili peppers. This study demonstrates the efficacy and tolerability of the sustained-intestinal release of CapF at both 100 and 200 mg/day doses (2.1 mg and 4.2 mg capsaicinoids/day) to enhance energy expenditure (resting and exercise-induced), respiratory quotient, fat oxidation, and endurance performance. These findings support the potential use of CapF as an effective and safe supplement for weight maintenance and as an ergogenic aid.

The significance of the study also comes from the observation that CapF at the tested dose was tolerated and did not produce adverse events such as increased heart rate, pulse rate, palpitation, sweating, and abdominal pain despite its efficacy. The slow-release mechanism protected the capsaicinoids from the stomach environment, thereby preventing gastrointestinal irritation commonly associated with the consumption of capsaicin or pungent red chili extracts.

## Data availability statement

The original contributions presented in the study are included in the article/supplementary material, further inquiries can be directed to the corresponding author.

## Ethics statement

The studies involving humans were approved by Sri Rama Hospital, Bangalore, India. The studies were conducted in accordance with the local legislation and institutional requirements. The participants provided their written informed consent to participate in this study.

## Author contributions

NR: Investigation, Writing – review & editing. DS: Data curation, Writing – original draft. IK: Conceptualization, Formal analysis, Methodology, Writing – review & editing. KM: Investigation, Writing – review & editing. BF: Writing – review & editing. JT: Investigation, Project administration, Writing – review & editing.
