# Corrigendum: A natural sustained-intestinal release formulation of red chili pepper extracted capsaicinoids (Capsifen^®^) safely modulates energy balance and endurance performance: a randomized, double-blind, placebo-controlled study

**DOI:** 10.3389/fnut.2024.1428397

**Published:** 2024-06-04

**Authors:** N. Roopashree, Das S. Syam, I. M. Krishnakumar, K. N. Mala, Bradley S. Fleenor, Jestin Thomas

**Affiliations:** ^1^BGS Global Institute of Medical Sciences, Bangalore, Karnataka, India; ^2^Akay Natural Ingredients Ltd, Kochi, Kerala, India; ^3^Sri Rama Hospital, Bangalore, Karnataka, India; ^4^DeBusk College of Osteopathic Medicine, Lincoln Memorial University, Harrogate, TN, United States; ^5^Leads Clinical Research and Bio Services Private Limited, Bangalore, Karnataka, India

**Keywords:** capsaicin, thermogenesis, Capsifen, endurance, energy expenditure, FenuMat, respiratory quotient

In the published article, there was an error in Figures 3A, C, 5B as published. In Figure 3A, there was an error in representation (second and third inset of Figure 3A were interchanged), but it was correctly mentioned in the results section. In Figure 3C, significance was marked wrongly in CapF 100 even though there is no significance. In Figure 5B, the percentage difference provided was wrong and the correct values were mentioned in results section. The corrected Figures 3A, C, 5B and its caption appear below.

**Figure 3:**
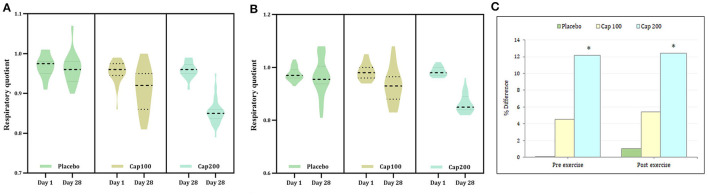
Violin plot showing the acute and chronic effects of CapF on respiratory quotient (RQ) at rest and at one hour following the maximal exercise for three groups (Placebo, CapF 100 and CapF 200). The violin plot outlines demonstrate kernel probability density. The width of the shaded area shows the distribution of the data. The thick dotted line shows the median, and thin dotted lines represent quartiles. **(A)** Treatment Vs time of RQ before exercise showed significant reduction at doses of 100 mg/day (*P* < 0.001) and 200 mg/day (*P* < 0.001) respectively at the end of study. The percentage of reduction were 4.2 and 11.45% respectively; **(B)** Treatment Vs time of RQ after exercise showed significant reduction at doses of 100 mg/day (*P* < 0.001) and 200 mg/day (*P* = 0.001) respectively at the end of study. The percentage of reduction were 5.10 and 12.24% respectively. The values are expressed as mean ± SD. **(C)** Percentage difference based on baseline values for Placebo, CapF 100 and CapF 200. The symbol “^*^” in the bar diagram indicate significant difference at *P* < 0.05 compared to placebo.

**Figure 5:**
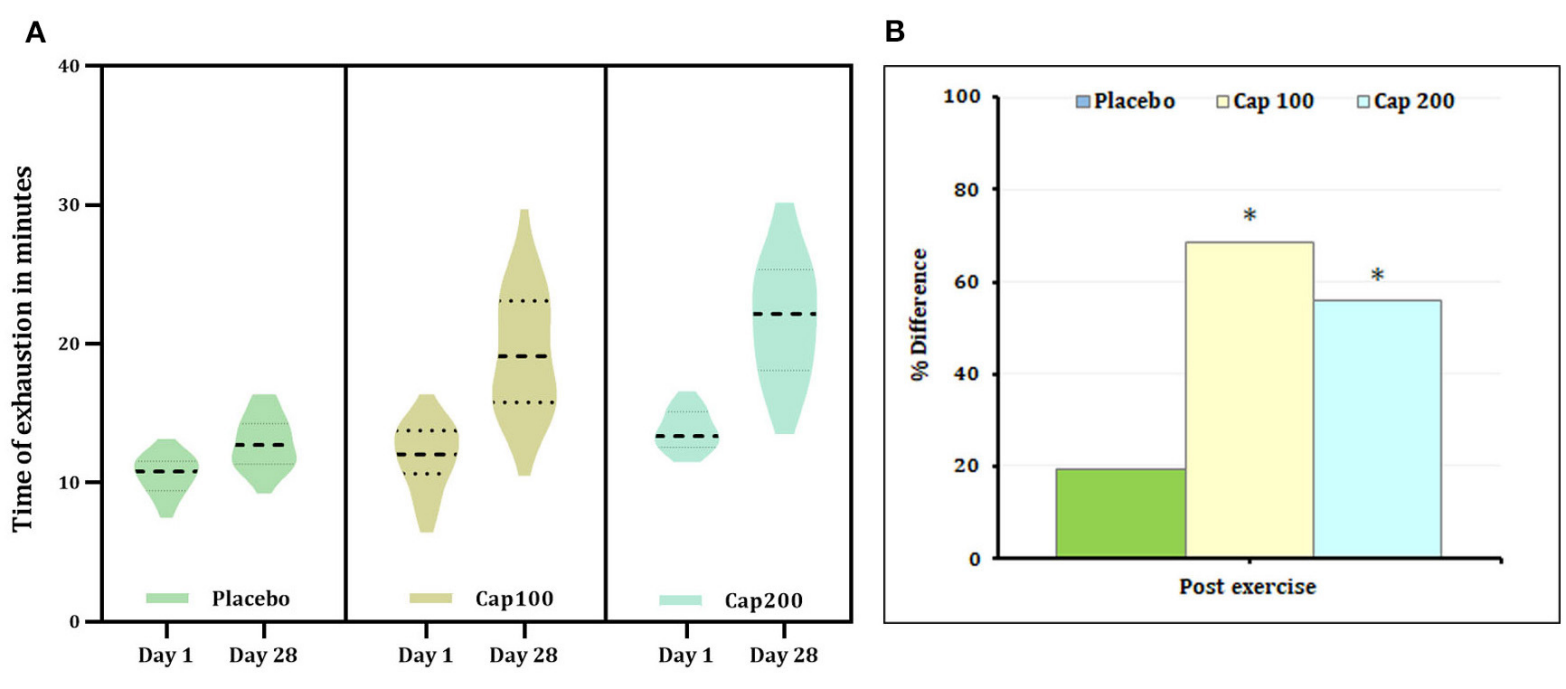
**(A)** Violin plot showing the acute and chronic effects of CapF on the time to exhaustion at rest and at one hour following the maximal exercise for three groups (Placebo, CapF 100, and CapF 200). The violin plot outlines demonstrate kernel probability density. The width of the shaded area shows the distribution of the data. The thick dotted line shows the median, and thin dotted lines represent quartiles. The results revealed significant increase in time of exhaustion compared to baseline upon supplementation with CapF 100 and CapF 200 (*P* < 0.001). The increase in CapF 100 is 68.52% (*P* < 0.001) and that of CapF 200 is 56.13% (*P* < 0.001). No significant change was observed in placebo (*P* > 0.05). **(B)** Percentage difference based on baseline values for Placebo, CapF 100 and CapF 200. The symbol “^*^” in the bar diagram indicate significant difference at *P* < 0.05 compared to placebo.

The authors apologize for this error and state that this does not change the scientific conclusions of the article in any way. The original article has been updated.

